# In-house ELISA screening using a locally-isolated *Leptospira*in Malaysia: determination of its cut-off points

**DOI:** 10.1186/s12879-014-0563-7

**Published:** 2014-10-23

**Authors:** Xue Ting Tan, Fairuz Amran, Kee Chee Cheong, Norazah Ahmad

**Affiliations:** Bacteriology Unit, Infectious Disease Research Centre, Institute for Medical Research, 50588 Jalan Pahang, Kuala Lumpur, Malaysia; Epidemiology and Biostatistics Unit, Medical Research Resource Centre, Institute for Medical Research, 50588 Jalan Pahang, Kuala Lumpur, Malaysia

**Keywords:** In-house ELISA, Leptospirosis, Malaysia, Cut-off points

## Abstract

**Background:**

Leptospirosis is a zoonotic disease caused by *Leptospira* species and is distributed globally. Microscopic agglutination test (MAT) is the serological ‘gold standard’ for diagnosis of leptospirosis but it is time-consuming and labour-intensive. An alternative serological method that is rapid, sensitive and specific is important for early treatment to reduce morbidity and mortality. The use of local *Leptospira* isolation may improve the sensitivity and specificity of the test because it may varies from one geographical region to another region. The objective of this study was to determine the sensitivity, specificity and cut-off points for an in-house Immunoglobulin M (IgM) enzyme-linked immunosorbent assay (ELISA) using a locally isolated Leptospiral strain IMR/175 as the antigen for the detection of anti-Leptospiral IgM.

**Methods:**

Serum samples from 270 patients with clinical symptoms of leptospirosis were subjected to the in-house IgM ELISA, MAT and Leptospirosis rapid test. The optimal cut-off values for positivity and negativity of the IgM ELISA were determined by Receiver Operating Characteristic curves and mean ± 2 standard deviation (SD) analyses of the ELISA values.

**Results:**

The area under the curve (AUC) which indicates the diagnostic performance of the in-house IgM ELISA was 0.953 (95% Confidence Interval, CI: 0.928, 0.978). The sensitivity and specificity of 90.38% and 87.72% respectively were obtained with the cut-off point of 0.55. A higher sensitivity (96.15%) was obtained when the cut-off point was set at 0.45.

**Conclusions:**

The in-house IgM ELISA assay using local *Leptospira* isolation was shown to be sensitive and may be suitable to use for the serological diagnosis of leptospirosis for our local hospital setting.

**Electronic supplementary material:**

The online version of this article (doi:10.1186/s12879-014-0563-7) contains supplementary material, which is available to authorized users.

## Background

Leptospirosis is an acute febrile disease with worldwide distribution [1],[2]. It is a problem of developing countries due to humid tropical and subtropical weather. It is identified as a re-emerging infectious disease since large outbreaks have occurred in many countries including Malaysia [3]. The overall incidence of leptospirosis in Malaysia was 13% [4] with fatality percentage is estimated around 10% [5].

Most of the human cases of leptospirosis are diagnosed by serological methods. The reference standard for serological test is the microscopic agglutination test (MAT) [5]. However, MAT has many disadvantages including time-consuming, tedious maintenance of live *Leptospira*, risk of cross-contaminations and hazardous to workers because of the exposure to live bacteria [6]. Besides that, MAT may not be useful for acute patient management because of the extensive technical resources requirements and is also not applicable in some routine diagnostic laboratory setting [7].

By comparison, the Immunoglobulin M (IgM) enzyme-linked immunosorbent assay (ELISA) is relatively cheaper than MAT and is more sensitive because of IgM can be detected earlier [6],[8]. This is important because treatment can be administered in time thus preventing the progression of the disease into severe stage.

Most of the commercially available ELISA kits using non-pathogenic *L. biflexa strain patoc I* as the antigen [6],[9]-[11]. Since the prevalence of Leptospiral serogroups varies geographically [12], we developed an in-house ELISA using a locally isolated *Leptospira* strain IMR/175 as the antigen for the detection of *Leptospira* IgM antibodies among patients in Malaysia. To our knowledge, this is the first study about in-house ELISA on detecting Leptospirosis in Malaysia. The aim of this study was to determine the cut-off points of the in-house ELISA and their accuracy, sensitivity and specificity.

## Methods

### Ethics statement

The study protocol which included participants providing written consent prior to the study, was approved by Malaysia Research & Ethics Committee, Ministry of Health Malaysia, Malaysia.

### Serum samples

A total of 270 sera received from patients presented with symptoms suggestive of leptospirosis during the year 2012 were subjected to a commercially available leptospirosis rapid test kit Leptorapid®, MAT, and in-house IgM ELISA in our laboratory. The usual presentations of the patients were acute febrile illness, headache, myalgia, jaundice, cough, vomit, abdominal pain, diarrhea and haemorrhages. The sample size was calculated based on expected sensitivity of 85%, specificity of 90%, precision of 0.10 and 95% of confidence level [4],[13].

### Leptospirosis rapid test

The rapid test was performed according to the instruction of Leptorapide® (Linnodee Ltd, Northern Ireland). Briefly, about 5 μl of each leptorapide suspension and test sera were mixed on the agglutination card. The agglutination was examined within three minutes and the results were interpreted using a score card provided by manufacturer.

### MAT

The MAT was performed as described by the World Health Organization [2]. Briefly, live *Leptospira* cell suspensions from 20 Leptospiral serovars were added to a two-fold serially diluted serum in 96-well U-bottomed microtiter plates and were incubated at room temperature for 2 hours. The panel of leptospires consisted of IMR/1, IMR/22, IMR/27, IMR/115, IMR, 175, IMR/803, Australis, Autumnalis, Bataviae, Canicola, Celledoni. Grippotyphosa, Hardjoprajitno, Icterohaemorrhagiae, Javanica, Pyrogenes, Tarassovi, Djasiman, Patoc and Pomona.

Micro-agglutination was examined by dark-field microscopy. The titer was calculated as the reciprocal of the highest dilution of serum which showed at least 50% of agglutination of the Leptospiral cells. Cases were defined as positive for leptospirosis if agglutination titer achieved ≥400 level to one or more *Leptospira* serovars. All samples were screened by MAT to determine the serum is positive or negative for leptospirosis.

### IgM ELISA

The antigen was prepared from a nonpathogenic strain IMR/175 which was isolated from a water sample in a pond from Sarawak, Malaysia. The antigen was diluted 1:2 in phosphate buffered saline (PBS) pH 7.2. The protocol of preparing antigen and coating plate was according to the protocol described by Goris *et al*. [14]. Briefly, the culture was grow in EMJH medium, killed by formalin (final concentration 0.2% v/v), heated in a boiling water bath for 30 min and centrifuged for 30 min at 10,000 g. The 100 μl supernatant was pipetted into the well and left to be evaporated. Before the plate being used, it was washed twice with PBS containing 0.05% Tween 20. Serial serum dilutions of 80 and 160 were added into the coated plates and incubated for 30 min at 37°C. The plate was then washed again and subsequently 100 μl of Horseradish peroxidase-conjugated anti-human IgM (KPL) was added to the plate. After 30 min incubation at 37°C, the plate was washed again. Next, 100 μl of substrate (5-aminosalicyclic acid) was added and the plate incubated for one hour at room temperature. The absorbance of the suspensions was read at 490 nm using Opsys Mr^TM^ Microplate reader (Dynex Technologies). The sample was tested in duplicates and an average optical density was obtained for each sample. Each reading was recorded and statistically analysed as mentioned below.

### Statistical analysis

All statistical analysis were performed with the statistical software package PASW statistics 18.0 for windows (SPSS Inc.). Receiver operating characteristic (ROC) curves were used to determine the optimal level of the IgM ELISA test for serum dilutions of 1:80 and 1:160. The sensitivities and specificities of IgM ELISA for diagnosis of leptospirosis (positive MAT) were also demonstrated at different cut-off values. In addition, the positive predictive value (PPV) and negative predictive value (NPV) were also calculated at different cut-off values.

Sensitivity is defined as the probability of correctly identifying those with positive leptospirosis by MAT for a given ELISA cut-off value. Specificity is defined as the probability of correctly identifying those with negative leptospirosis by MAT at a given IgM ELISA cut-off value. PPV is the probability that subjects with a positive ELISA test truly have leptospirosis while NPV is the probability that subjects with a negative ELISA test truly do not have leptospirosis.

The optimal IgM ELISA cut-off value was determined by comparing the mean ± 2 standard deviation (SD) of IgM ELISA value for both positive and negative MAT samples. The area under the curve (AUC) with 95% confidence intervals was used to compare the predictive capability of the two IgM ELISA dilutions for identification of those diagnosed by MAT. Higher of the values of AUC indicates a higher predictive capability of IgM ELISA in diagnosing leptospirosis. For all analyses, *p* values less than 0.05 were considered as statistically significant.

## Results and discussion

Out of 270 samples tested, 114 samples were negative while 156 were positive by both MAT and rapid test. The samples were further tested by in-house ELISA and all the obtained data were used for ROC curve analysis to determine the cutoff point from optimal sensitivity and specificity achieved in the assay.

**Figure 1:**
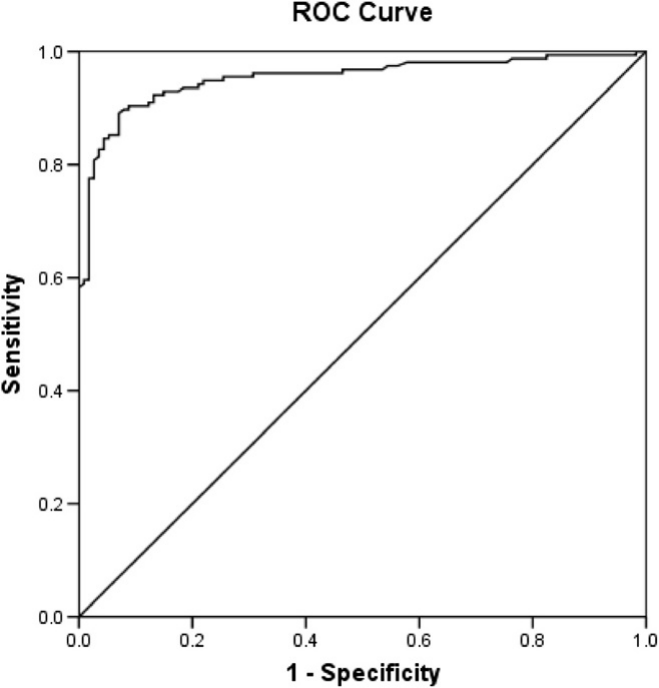
**ROC demonstrating the sensitivity and 1-specificity for diagnosis of leptospirosis at various IgM ELISA points for dilution 1:80.**

**Figure 2:**
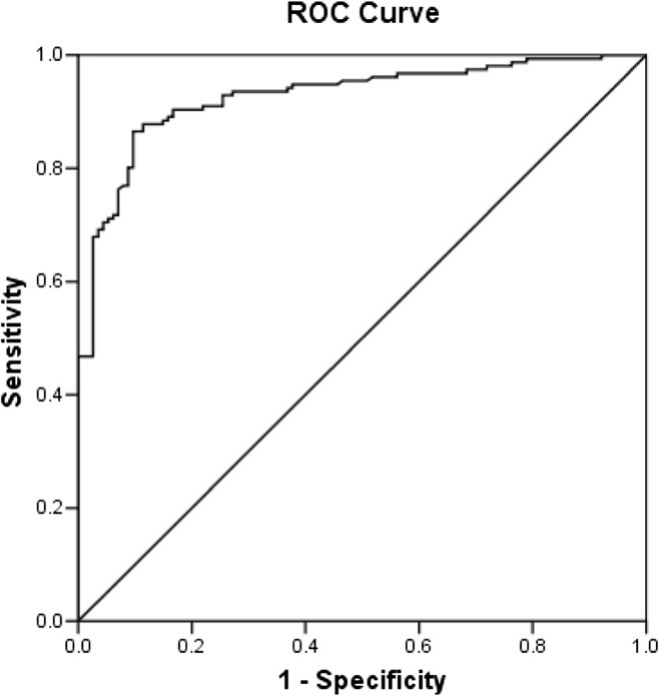
**ROC demonstrating the sensitivity and 1-specificity for diagnosis of leptospirosis at various IgM ELISA points for dilution 1:160.**

The AUC reflects how good of the test to distinguish between patients with and without disease [14]. In the ROC curve analysis, it showed that the AUC for serum dilutions 1:80 and 1:160 were 0.953 (95% Confidence Interval, CI =0.928-0.978) and 0.927 (95% CI =0.896-0.958) respectively (Figures 1 and 2). Although the diagnostic performances of our in-house IgM ELISA were high for both dilutions, the 1:80 dilution had better diagnostic performance than 1:160. Thus, we decided to select cut-off points from the 1:80 dilution.

The mean ± 2SD value for both positive and negative MAT samples of dilutions are listed in Table 1. To determine the negative and positive cut-off points, we referred to the value of mean-2SD of positive MAT samples and mean + 2SD of negative MAT samples respectively. After that, the sensitivity and specificity of yielded cut- off values from ROC curves analysis were compared (Table 2). From the analysis of mean ± 2SD of the 1:80 dilution, cut-off values of 0.45 and 0.64 were obtained. However, 0.64 was not selected as our positive cut-off point because it gave sensitivity of less than 90%. As the leptospirosis incidence and fatality of Leptospirosis in Malaysia is high, a very sensitive test is needed because it is very important not to miss a diagnosis [15]. We decided the sensitivity of the positive cut-off shall be equal or greater than 90%, thus 0.45 and 0.55 were selected as negative and positive cut-off points respectively. The value between 0.45 and 0.55 is considered intermediate level and it is recommended to be repeated with follow-up sample.

**Table 1:** **Statistical values of both positive and negative MAT samples for ELISA with serum dilutions of 1**:**80**

Statistical value	Positive MAT samples	Negative MAT samples
Mean	0.7783	0.4316
SD	0.1627	0.1057
Mean + 2 SD	1.1037	0.6430
Mean-2 SD	0.4529	0.2202

**Table 2:** **Sensitivity and specificity of IgM ELISA for dilution 1**:**80 and 1**:**160 at different cut**-**off values**

Dilution	Cut-off value	Sensitivity (%)	Specificity (%)	Positive predictive value (%)	Negative predictive value (%)
	0.45	96.15	57.89	75.76	91.67
1:80	0.50	94.87	75.44	84.09	91.49
	0.55	90.38	87.72	86.96	90.97
	0.60	85.26	93.86	95.00	82.31
	0.65	80.13	97.37	97.66	78.17
	0.45	88.46	84.21	88.46	84.21
1:160	0.50	83.33	90.35	92.20	79.84
	0.55	72.44	92.98	93.39	71.14
	0.60	66.67	97.37	97.20	68.10
	0.65	57.05	97.37	96.74	62.36

To emphasize, our institute is the main institute that carry out the leptospirosis routine diagnosis work which submitted from clinics and hospitals throughout the country [16]. It is important to note that about 50% of the positive sera were agglutinated with this strain in our MAT test yearly. We had submitted the partial sequence of 16S ribosomal RNA gene of this strain to NCBI (GeneBank: JX292159) and research on this strain is still ongoing.

As convalescent-phase serum is hardly available in Malaysia hospital, it is difficult to determine the fourfold or greater rise in MAT titers between acute- and convalescent-phase which define a leptospirosis case. Thus, we referred the guidelines given by Ministry of Health Malaysia [17]. The case was defined positive if single serum titre was equal to or greater than 400 while fourfold or greater rise in MAT titre if paired sera were obtained. The same case definition also used in other studies [11],[18]-[20]. Besides, the case also can be defined positive if MAT is greater than or equal to 400 and supported by a positive IgM result [21]. In this study, we had further increased the reliability of our result with including the samples which showed positive from both rapid and MAT tests. Lastly, further study on more clinical samples is necessary to determine the usefulness of our IgM ELISA.

## Conclusions

This study showed that using the cut-off points determined from 1:80 dilution in our in-house ELISA using local Leptospira isolation is sensitive for the detection of leptospirosis cases when compared to the MAT results. The positive and negative cut-off points for this IgM ELISA are 0.55 and 0.45 respectively.

## Authors’ original submitted files for images

Below are the links to the authors’ original submitted files for images. Authors’ original file for figure 1 Authors’ original file for figure 2

## Abbreviations

AUC: Area under the curve
CI: Confidence interval
ELISA: Enzyme-linked immunosorbent assay
IgM: Immunoglobulin M
MAT: Microscopic agglutination test
PBS: Phosphate buffered saline
ROC: Receiver operating characteristic
PPV: Positive predictive value
NPV: Negative predictive value
SD: Standard deviation
