# Supplementary figures and images for: In-house ELISA screening using a locally-isolated Leptospirain Malaysia: determination of its cut-off points
Source: BMC Infect Dis. 2014 Oct 23;14:563. doi: 10.1186/s12879-014-0563-7 (PMC4212092; doi:10.1186/s12879-014-0563-7)

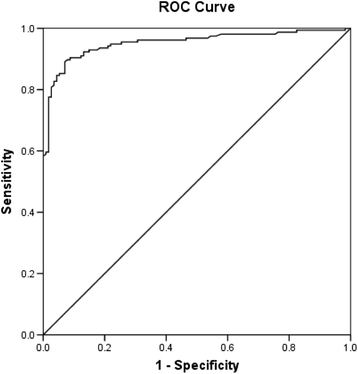

Supplement: Supplementary file 1 — Authors’ original file for figure 1 [file 12879_2014_563_MOESM1_ESM.gif]

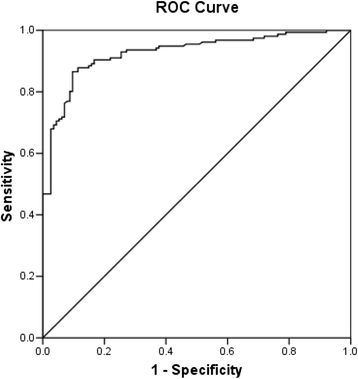

Supplement: Supplementary file 2 — Authors’ original file for figure 2 [file 12879_2014_563_MOESM2_ESM.gif]
